# Mutations and Deletions in PCDH19 Account for Various Familial or Isolated Epilepsies in Females

**DOI:** 10.1002/humu.21373

**Published:** 2011-01

**Authors:** Christel Depienne, Oriane Trouillard, Delphine Bouteiller, Isabelle Gourfinkel-An, Karine Poirier, François Rivier, Patrick Berquin, Rima Nabbout, Denys Chaigne, Dominique Steschenko, Agnès Gautier, Dorota Hoffman-Zacharska, Annie Lannuzel, Marilyn Lackmy-Port-Lis, Hélène Maurey, Anne Dusser, Marie Bru, Brigitte Gilbert-Dussardier, Agathe Roubertie, Anna Kaminska, Sandra Whalen, Cyril Mignot, Stéphanie Baulac, Gaetan Lesca, Alexis Arzimanoglou, Eric LeGuern

**Affiliations:** AP-HP, Département de génétique et cytogénétique, Fédération de Génétique, Hôpital de la Pitié-SalpêtrièreF-75013, Paris, France; INSERM, CRicm (U975), Hôpital de la Pitié-SalpêtrièreF-75013, Paris, France; UPMC Univ Paris 06F-75005, Paris, France; Pôle d'Epileptologie, Hôpital de la SalpêtrièreF-75013, Paris, France; Centre de référence épilepsies rares, Inserm U567, UMR 8104, Université René DescartesParis V, France; Institut Cochin, Inserm U567, UMR 8104, Université René DescartesParis V, France; CHU Montpellier, Service de Neuropédiatrie, Hôpital Gui de ChauliacMontpellier, F-34000 France; Service de neuropédiatrie, CHU Hôpital Nord AmiensAmiens, France; Département de Neuropédiatrie, AP-HP, Hôpital Necker-Enfants maladesParis-Descartes, Paris, France; Service de Neuropédiatrie - Clinique Sainte-OdileStrasbourg, France; Unité de Neurologie Pédiatrique, Hôpital d'EnfantsCHU de Nancy; Clinique Médicale Pédiatrique, Hôpital Mère-EnfantCHU de Nantes, France; Institute of Mother and Child Department of Medical GeneticsWarsaw, Poland; Department of neurology, University Hospital of Pointe-à-PitreGuadeloupe, F.W.I; Unit of genetics, university hospital of Pointe a PitreGuadeloupe, F.W.I; Service de NeuropédiatrieCHU de Bicêtre, Le Kremlin Bicêtre, France; Service de neuropédiatrie, Hôpital Mère-EnfantCHU de Nantes, France; Service de Génétique, Centre de Référence Anomalies du Développement de l'OuestCHU Poitiers, France; Service de Neuropédiatrie, CHU Montpellier, Hôpital Gui de Chauliac, and INSERM U827Montpellier, France; Service de Neuropédiatrie, Hôpital TrousseauParis, France; Service de génétique, University Hospitals of Lyon (HCL)Lyon, France; Institute for children and adolescents with Epilepsy IDEE, University Hospitals of Lyon (HCL)Lyon, France; Inserm U821France

**Keywords:** PCDH19, Epilepsy, Febrile seizures, microdeletion, cognitive function

## Abstract

Mutations in PCDH19, encoding protocadherin 19 on chromosome X, cause familial epilepsy and mental retardation limited to females or Dravet-like syndrome. Heterozygous females are affected while hemizygous males are spared, this unusual mode of inheritance being probably due to a mechanism called cellular interference. To extend the mutational and clinical spectra associated with PCDH19, we screened 150 unrelated patients (113 females) with febrile and afebrile seizures for mutations or rearrangements in the gene. Fifteen novel point mutations were identified in 15 female patients (6 sporadic and 9 familial cases). In addition, qPCR revealed two whole gene deletions and one partial deletion in 3 sporadic female patients. Clinical features were highly variable but included almost constantly a high sensitivity to fever and clusters of brief seizures. Interestingly, cognitive functions were normal in several family members of 2 families: the familial condition in family 1 was suggestive of Generalized Epilepsy with Febrile Seizures Plus (GEFS+) whereas all three affected females had partial cryptogenic epilepsy. These results show that mutations in PCDH19 are a relatively frequent cause of epilepsy in females and should be considered even in absence of family history and/or mental retardation. © 2010 Wiley-Liss, Inc.

## INTRODUCTION

Mutations in the PCDH19 gene, located on chromosome X and encoding the protocadherin 19, were originally identified in Epilepsy and mental retardation limited to females (or Epilepsy, Female-restricted, with Mental Retardation, EFMR; MIM# 300088) (Dibbens, et al., 2008). EFMR is a disorder with a remarkable X-linked inheritance: only females with heterozygous mutations are affected whereas males with hemizygous mutations are unaffected (Fabisiak and Erickson, 1990; Juberg and Hellman, 1971; Ryan, et al., 1997). This unusual mode of inheritance is likely to be due to cellular interference, a mechanism assuming that only the co-existence of PCDH19-positive and -negative cells, as a result of random X inactivation in females, is pathogenic (Depienne, et al., 2009; Johnson, 1980; Wieland, et al., 2004).

The phenotype of EFMR varies from mild to severe in terms of seizure type and severity as well as in the degree of cognitive impairment (Scheffer, et al., 2008). Seizures begin in infancy or early childhood (6–36 months) and are sensitive to fever in most patients. Patients present with variable seizure types including tonic-clonic, tonic, atonic, absences, myoclonic jerks and partial seizures. Behavioral problems are often part of the clinical picture and can manifest as autistic, obsessive or aggressive features (Depienne, et al., 2009; Scheffer, et al., 2008). Recently, we have shown that the clinical spectrum associated with PCDH19 mutations can overlap that of Dravet syndrome (Depienne, et al., 2009). Intellectual outcome was reported to range from normal intellect to mental retardation within families; however, all patients reported to date either had intellectual disability or belonged to families where cognitive delay was present in other family members (Scheffer, et al., 2008).

In the present study, we have screened 150 unrelated probands with diverse epilepsy subtypes with or without cognitive impairment or mental retardation in order to define the mutational and clinical spectra associated with PCDH19 mutations.

## MATERIALS AND METHODS

### Patients

We selected 150 unrelated probands (113 females and 37 males) for *PCDH19* screening. The referral physicians filled a detailed clinical questionnaire for every patient and clinical reports were also collected for most patients to assess the clinical history of the disease. Ninety-five percent (n=142) of the patients had been initially referred to our diagnosis laboratory for genetic testing of *SCN1A* based on the association of febrile and afebrile seizures. Most patients (n=104) had no family history of epilepsy. These sporadic cases include 16 patients with DS in whom *SCN1A* testing was negative. Forty-six probands had at least one relative with epilepsy. The mode of inheritance was compatible with that of EFMR in 10 families. Eight families were reported as GEFS+ (Generalized Epilepsy with Febrile Seizures Plus) conditions. Eight families had a homogeneous phenotype of generalized idiopathic epilepsy (n= 5) or partial epilepsy (n= 3). Informed written consent was obtained from the patients' parents before blood sampling. This study was approved by the ethical committee (CCPPRB of Pitié-Salpêtrière Hospital, Paris, n°69-03, 25/9/2003).

### PCDH19 Screening

The coding sequence of *PCDH19* (transcript reference EF676096) was amplified in 11 fragments, and screened using direct sequencing, as already described (Depienne, et al., 2009). Mutations found in patients were directly searched for in available parents using direct sequencing of the corresponding amplicon. When the mutation was absent from both parents, parental testing was performed using microsatellite markers at the *PCDH19* locus to ensure that the mutation occurred *de novo.* In addition, 190 French controls (95 females and 95 males who were healthy spouses of patients with other autosomal dominant diseases) and 200 Polish controls (118 females and 82 males) were included to test new variants of the *PCDH19* gene. Mutation interpretation and amino acid conservation in orthologs and paralogs was assessed using the Alamut 1.31 Software (Interactive Biosoftware) and Clustalw (available at http://bioinfo.hku.hk/services/analyseq/cgi-bin/clustalw_in.pl).

Deletions or duplications involving *PCDH19* were searched for using quantitative real-time PCR. Six primer pairs (one per exon) were designed using PrimerExpress 1.5 (sequences available on request). Real-time PCR experiments were performed using 100 ng of genomic DNA, 0.4 μM of each primer and 12.5 μl of Sybr Green PCR master mix (Applied Biosystems) in a total volume of 25 μl. The RNAse P (RNAse P control assay from Applied Biosystems) was used as the reference amplicon. Each sample was run in triplicate on an ABI PRISM 7900 Detection system (Applied Biosystems). Relative ratios were calculated using the formula r = 2^−ΔΔCt^ with ΔΔCt = (Ct_Mutation_ - Ct_RNAseP_)_ind tested_ – (Ct_Mutation_ – Ct_RNAseP_)_ind ref_

### Characterization of *PCDH19* deletions using high density SNP arrays

Patients with *PCDH19* deletions were analyzed using 370CNV-Duo genotyping BeadChip arrays (Illumina). The Infinium II Genotyping reaction steps were performed according to the manufacturer's specifications. Image data analysis and automated genotype calling was performed using Beadstudio 3.1 (Illumina). Breakpoints of the deletions were defined as the first and last SNP comprised in the region of the deletion, which were homozygous and presented a decreased log ratio (in the order of -0.5).

## RESULTS

### Genetic analyses

**Figure 1:**
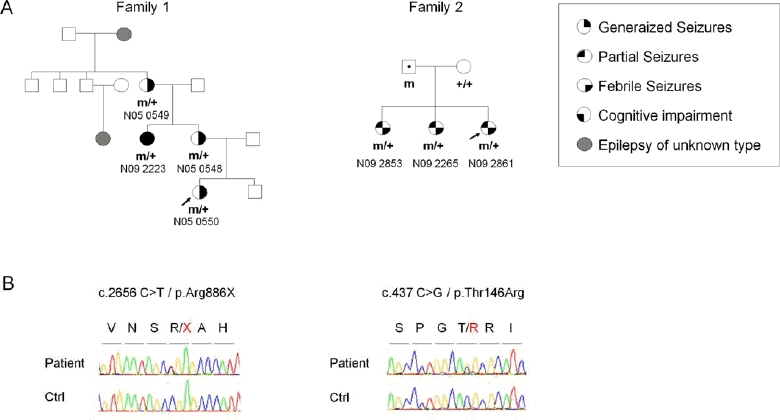
Identification of 2 families with PCDH19 mutations, in which the majority of patients had normal cognitive functions. A) Pedigrees of the families and segregation analysis of the PCDH19 mutations; m/+ and m: individuals heterozygous and hemizygous for the mutation; +/+ and +: individuals homozygous and hemizygous for the wild-type allele. Dots in the middle of the squares: unaffected mutation carrier. The arrows indicate the index cases. B) Sequence electrophoregrams of the mutations. Mutation nomenclature is based on the PCDH19 transcript reference EF676096. Nucleotides are numbered according to the cDNA with +1 corresponding to the A of the ATG translation initiation codon in the reference sequence.

One hundred and fifty unrelated probands (113 females and 37 males) were screened for mutations in *PCDH19* by direct sequencing. A total of 15 different mutations, all novel, were identified at the heterozygous state in 15 female patients (Tables 1A, Figure 1 and Supp. Fig. S1). Eight were missense variants (c.242T>G/p.Leu81Arg; c.437C>G/p.Thr146Arg; c.617T>A/p.Phe206Tyr; c.747A>T / p.Glu249Asp; c.1023C>G/p.Asp341Glu; c.1682C>G/p.Pro561Arg; c.1700C>T/p.Pro567Leu; C.1852G>A/ p.Asp618Asn), 2 were nonsense mutations (c.462C>A/ p.Tyr154X; c.2656C>T/ p.Arg886X), 4 were base pair insertions or deletions introducing a frameshift into the protein sequence (c.424delG/ p.Ala142ProfsX70; c.514dupG/p.Glu172GlyfsX54; c.697_700delinsTAAC / p.Asp233X; c.2019delC / p.Ser674LeufsX2) and one was an in-frame duplication of three amino-acids (c.415_423dup/p.Ser139_Ala141dup). All mutations were located in exon 1 with the exception of the p.Arg886X nonsense mutation, which was located in exon 4. Missense variants all affected amino-acids of the extracellular domain of protocadherin 19, which are highly conserved in orthologs and in paralogs of *PCDH19* (Supp. Fig. S2). To confirm that the 15 mutations were pathogenic, we screened 190 healthy French individuals. None was found in the control population, supporting their deleterious role. Since Family 2, in which the c.437C>G /p.Thr146Arg mutation was identified, was of Polish origin, this variant was also tested and not found in 200 healthy Polish Controls.

Parents of mutated probands and affected relatives were tested for the mutation when available. Six out of the 15 probands with *PCDH19* mutations were isolated cases (families 3, 5, 6, 11, 13 and 14) but both parents were available in only 3 families. The mutation was absent in both parents in one family out of the 3, indicating that it arose *de novo* (family 11). Remarkably, in the 2 other families (13 and 14), the mutation was inherited from the asymptomatic mother. In familial cases (families 1, 2, 4, 7, 8, 9, 10, 12 and 15), mutations were inherited from a healthy father in 3 families (families 2, 9 and 12), leading to a recurrent transmission of the mutation to all the daughters. In family15, which yet include two affected daughters, the mutation was absent from both parents, suggesting germinal mosaicism in one parent. However, the sister of the proband was unavailable for genetic analysis, so it remains uncertain whether she had the same mutation than the proband. In families 1, 4 and 10, the mutation was inherited from an affected mother and segregated with the disease in all affected family members. It was not possible to determine the origin of the mutation in the remaining families (families 3, 5, 6, 7, and 8) although the presence of an affected maternal aunt in family 7 argued in favour of paternal inheritance.

**Table 1A:** Female patients with point mutations identified by direct sequencing

Family	Patients (proband)	Exon	Amino acid changes	Consequence at the protein level	Parents' analysis	Associated polymorphisms
3	N 09 0176	1	c.242T>G	p.Leu81Arg	Unknown	none

4	N 09 0418	1	c.415_423dup	p.Ser139_Ala141dup	Maternal inheritance	none

5	N 09 1326	1	c.424delG	p.Ala142ProfsX70	Unknown	none

2	N09 2861	1	c.437C>G	p.Thr1 46Arg	Paternal inheritance	c.1627C>T/p.Leu543Leu

6	N 09 1568	1	c.462C>A	p.Tyr154X	Unknown	none

7	N 09 1461	1	c.514dupG	p.Glu172GlyfsX54	Unknown (paternal inheritance expected)	none

8	N 05 1365	1	c.617T>A	p.Phe206Tyr	Unknown	c.3447+8 T>C

9	N 08 1081	1	c.697_700delinsTAAC	p.Asp233X	Paternal inheritance	none

10	N 08 1391	1	c.747A>T	p.Glu249Asp	Maternal inheritance	c.1627C>T/p.Leu543Leu

11	N 09 1071	1	c.1023C>G	p.Asp341 Glu	De novo	none

12	N 08 0696	1	c.1682C>G	p.Pro561Arg	Paternal inheritance	none

13	N 09 1090	1	c.1700C>T	p.Pro567Leu	Maternal inheritance	none

14	N 08 1063	1	c.1852G>A	p.Asp618Asn	Maternal inheritance	c.1627C>T/p.Leu543Leu

15	N 09 1135	1	c.2019delC	p.Ser674LeufsX2	De novo (mosaicism in one parent?)	none

1	N 05 0550	4	c.2656C>T	p.Arg886X	Maternal inheritance	c.1627C>T/p.Leu543Leu

**Figure 2:**
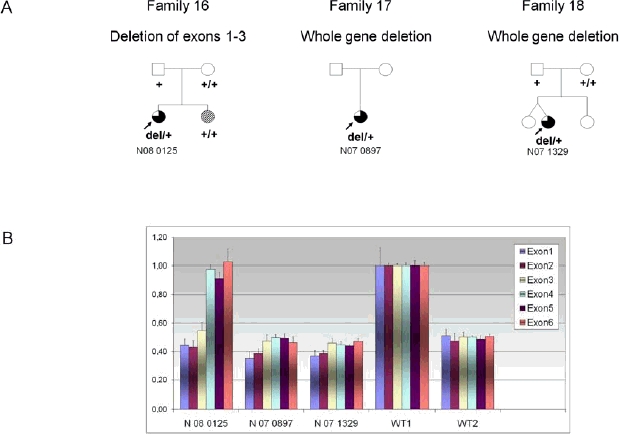
Identification of 3 families with PCDH19 deletions. A) Pedigrees of the families and segregation analysis of the PCDH19 deletions; del/+: individuals heterozygous for the deletion; +/+ and +: individuals homozygous and hemizygous for the wild-type allele, respectively. Upper right black corner: Generalized seizures; Upper left black corner: Partial seizures; Lower right black corner: FS; Lower left black corner: intellectual disability; hatched symbols: patients with rolandic epilepsy. The arrows indicate the index cases. B) Results of copy number dosage for each exon of PCDH19 using real time-Q-PCR. WT1 is a control female; WT2 is a control male.

**Figure 3:**
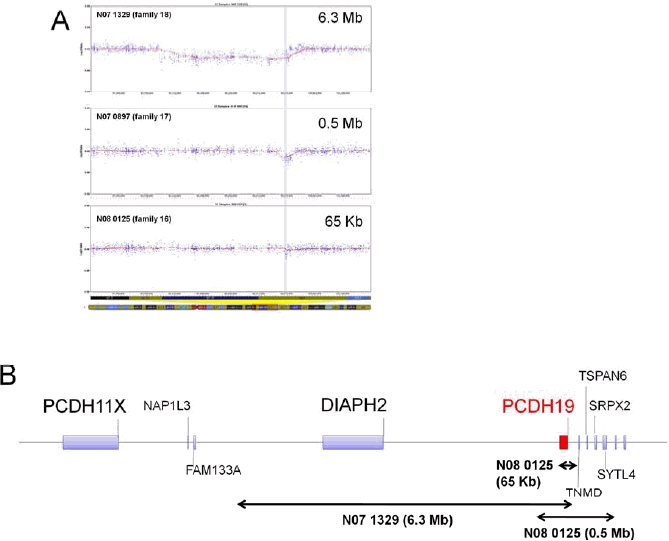
Characterization of the size and breakpoints of the PCDH19 deletions using high-density SNP arrays (Illumina). A) Log R profiles of the 3 patients with deletions. The X-axis indicates the position on the chromosome X and the Y-axis indicates the log R. The grey stripes represent the location of the PCDH19 gene. B) Schematic representation of the deletions indicated by double arrows. The size of the deleted regions (in Mb of Kb) is indicated in brackets.

In order to uncover micro-rearrangements in *PCDH19* that could have been missed by direct sequencing, we developed quantitative real-time PCR assays to measure the copy number of each exon of the gene. This analysis identified two whole gene deletions and a partial deletion spanning exons 1 to 3, in 3 additional female patients (Figure 2 and Table 1B). Analysis of the corresponding patients' DNA using high density SNP array revealed that the deletions had different size and breakpoints. The whole gene deletions in patients N07 0897 (family 17) and N07 1329 (Family 18) spanned 0.5 and 6.3 Mb, respectively, and comprises nearby genes (Figure 3). The largest deletion encompasses DIAPH2, a gene possibly involved in oogenesis and associated with premature ovarian failure type 2 (Bione, et al., 1998). The 0.5 Mb deletion includes TNMD, TSPAN6, SYTL4 and SRPX2, the gene responsible for X-linked recessive rolandic seizures associated with oral and speech dyspraxia and mental retardation (Roll, et al., 2006). Contrary to the two other deletions, deletion of exons 1 to 3 was limited to the *PCDH19* gene in patient N08 0125 (family 16). Parents of the proband were unavailable in family 17 but analysis of the parents in families 16 and 18 revealed that the deletions arose *de novo*.

**Table 1B:** Female patients with deletions identified by semi-quantitative real-time PCR

Family	Patients	Exon	Quantitative PCR	Consequence at the protein level	Parents' analysis	Associated polymorphisms
16	N 08 0125	1 to 3	deletion of exons 1 to 3	Absence of protein synthesis?	De novo	none

17	N 07 0897	1 to 6	Whole gene deletion	Absence of protein synthesis	Unknown	none

18	N 07 1329	1 to 6	Whole gene deletion	Absence of protein synthesis	De novo	none

### Summary of clinical features for PCDH19-positive patients

The clinical features of *PCDH1* 9-positive patients are summarized in Table 2. The clinical pictures were variable from one patient to another. However, seizures were highly sensitive to fever in most patients, especially in the 2 first years of life and during childhood. Another characteristic feature observed in *PCDH19*-positive patients was seizures occurring in clusters (2-10 seizures per day, lasting over several days). Remarkably, cognitive functions ranged from normal to severe intellectual deficiency. Indeed, several or all family members of 2 families (families 1 and 2) had no cognitive impairment.

**Table 2:** Clinical features of patients with PCDH19 mutations or deletions

Family	Patient Number	Age at exam (years)	Age at first seizure (months)	Type of the first seizure	Type of seizures	SE	Sz in cluster	Myocl.	Pho Sen.	Sensiti vity to fever	Ataxia	IDorPD	Language	AEDs (+)	AEDs without effects	AEDs (−)	Others
1	N05 0550	6	11	Febrile GTCS	Febrile and afebrile GTCS Absences	no	yes	no	no	+	no	none	normal	PB	VPA	−	−

1	N05 0548	28	18	Febrile GTCS	Febrile and afebrile CTGC, absences	no	yes	no	no	++	no	none	normal	PB	VPA	−	−

1	N05 0549	54	18	GTCS	GTCS, absences	no	yes	yes	NA	NA	no	none	normal	PB	VPA	−	−

1	N09 2223	36	18	Prolonged febrile GTCS	Febrile and afebrile, GTCS, loss of consciousness (absences or partial sz?)	Yes (first sz)	yes	no	no	++	no	moderate	normal	PB, VPA	−	CBZ	Behaviour disturbances

2	N09 2853	24	7	Febrile GTCS	GTCS Partial sz	no	yes	no	no	+++	no	none	normal	CBZ, LEV	VPA, VGB, LTG, TPM	−	−

2	N09 2265	23	60	Febrile GTCS	GTCS Partial sz	no	yes	no	no	+++	no	none	normal	CBZ	VPA	−	−

2	N09 2851	15	9	afebrile GTCS	GTCS Partial sz	no	yes	no	No	+++	no	none	normal	LTG,LEV	CBZ	−	−

3	N09 0176	12	11	Febrile GTCS	GTCS	yes	yes	NA	NA	+++	?	mild	words-sentences	VPA, CLB	−	LTG	Behaviour disturbances

4	N09 0418	7	13	Febrile GTCS	GTCS	no	yes	no	no	+++	no	mild	Delayed (sentences)	CLN, TPM,	VPA	−	Behaviour disturbances

5	N09 1326 (DS)	20	10	Afebrile prolonged GTCS	Febrile and afebrile GTCS partial sz	yes	yes	no	no	+++	no	moderate	words-sentences	TPM, LEV, ZNS	CBZ, PB, VPA, LTG		Behaviour disturbances

6	N09 1568	9	13	NA	GTCS	no	yes	no	no	+++	no	Very mild	NA	VPA, CLN	−	−	−

7	N09 1461	1	8	afebrile GTCS	GTCS	no	yes	NA	NA	NA	too young	too young	too young	NA	VPA, PHT	−	Slight dysmorphic features

8	N05 1365	13	60	afebrile	Hemiclonic, Partial sz, Absences	yes	yes	no	no	+	yes	moderate	sentences	LTG, CBZ	VPA	−	−

9	N09 1515	14	24	Febrile Complex partial hemi clonic, generalised	Motor complex partial 2nd generalised	yes	yes	no	no	++	subtle	moderate	sentences	LEV, TPM	VGB	LTG	Behaviour disturbances

9	N09 1516	13	8	Febrile	Motor complex partial 2nd generalised	yes	yes	no	no	++	subtle	moderate	sentences	LEV, TPM,CLN	VGB	−	−

10	N08 1391	7	8	febrile, generalized, in cluster	Generalized sz Partial sz	no	yes	no	no	+++	yes	moderate	words-sentences	PB, TPM, CLN, LEV STP	LTG, CBZ, VPA	VGB	Fatiguability

11	N09 1071	10	NA	NA	Partial sz GTCS	NA	yes	no	NA	NA	NA	mild	words-sentences	CLN, LTG, PB	−	−	−

12	N08 0696	4	11	febrile, GTCS	GTCS, Absences Partial sz	yes	no	no	no	++	no	mild	words-sentences	VPA, CLB	−	−	−

12	N091103	2.5	8.5	afebrile, generalized tonic	atypical absences, tonic, GTCS	no	no	no	no	no	no	mild	words-sentences	TPM, VPA, CLN	−	−	acquired microcephaly

13	N091090	14	15	Febrile GTCS	brief febrile GTCS, atypic absences Myoclonic jerks	No but prolon ged sz(15 min)	yes	yes	yes	+++	yes	moderate/ severe	sentences	VPA, LTG, CLB, LEV?	−	−	−

14	N081063	2	8	febrile, GTCS	Clonic sz Atonic seizures	no	yes	no	no	no	too young ?	moderate	less than 10 isolated words at 2y	VPA, CLB	−	−	Delayed fine motor skills, Delayed myelination

15	N09 1135	6	9	afebrile, generalized	GTCS	yes	yes	no	no	+++	yes	moderate	words-sentences	VPA, CLB, LTG	TPM	CBZ	−

16	N08 0125	17	9	Generalized	Generalized	yes	yes	no	no	+++	no	moderate/s evere	?	VGB, TPM, NTZ	−	−	Behaviour disturbances

17	N07 0897	7	15	febrile, generalized, in cluster	Generalized Hypertonic	no	yes	no	NA	+++	no	moderate	words-sentences	VPA, TPM,CLB	PB, PHT	−	−

18	N071329	11	14	Afebrile generalized	GTCS	Yes (first sz)	yes	no	no	+++	no	moderate	normal	VPA, TPM, CLB, LTG, CLN	−	−	Delayed fine motor skills, Behaviour disturbances

SE: *Status Epilepticus* ; ID: Intellectual deficiency; PD: psychomotor delay ; Sz: Seizures; Myocl. : Myoclonic jerks ; Pho Sen. : Seizure photosensitivity ; AEDS : Antiepileptic drugs ; AEDs (+): AEDs with positive response ; wo: without; AEDs(−): AEDs with negative response (worsening of the seizures or of the patient state); GTCS : Generalized tonic clonic seizures ; NA: Not available ; CBZ : Carbamazepine, CLB: Clobazam, CLN: Clonazepam , LEV : Levetiracetam, LTG: Lamotrigine, NTZ: Nitrazepam, PB: Phenobarbital, PHT: phenytoin, STP : Stiripentol, TPM : topiramate, VGB: Vigabatrin, VPA : Sodium Valproate, ZNS: zonisamide.

### Case reports

Family 1, of French origin, includes 4 female patients. Neonatal periods were uneventful for all patients. The proband (N05 0550) was a 6-year-old girl. Her affected mother was treated with Phenobarbital (PB) during her whole pregnancy. GTCS, lasting ∼20 seconds and occurring in clusters of 5-10 seizures per day, have appeared at 11 months of age, after a febrile episode. Clusters of seizures have persisted for 10 days. Neurological status of the patient was normal between seizures. Clinical investigations, including search for infectious agents, lumbar puncture and CT scan, were normal. Seizures persisted but became rarer when the patient was treated with sodium valproate (VPA) and Clobazam (CLB), and finally stopped with PB. Later on, GTCS became very rare, occurring most often in afebrile contexts, but absence episodes were sometimes observed. EEGs were normal. Psychomotor development of the patient was normal and she had no school difficulties at 6 years of age. She was still treated with PB at the time of the study. Her mother (N05 0548) was 28 years old at the time of the study. At 18 months of age, while she was febrile, she presented a cluster of 4-5 brief GTCS per day that lasted for several days. Later on, febrile or afebrile GTCS regularly recurred until age 7. VPA appeared inefficient but GTCS finally ceased when she was treated with PB. However, absence episodes, mostly triggered by fatigue were observed. EEGs and cerebral MRI were normal. The maternal grandmother of the proband (N05 0549) was 54 years old when examined for the last time. As her daughter, she has had a cluster of GTCS lasting several days at 18 months of age although it remained uncertain whether she was febrile or not at this time. GTCS persisted until age 10, and then absences occurred from age 15. Myoclonic jerks were also noticed during adolescence. VPA was inefficient but PB allowed a reasonable control of the seizures. She was still treated with PB at the time of the study. Her psychomotor development had always been normal and no cognitive deficit was perceptible. EEGs and cerebral MRI remained unavailable. The maternal aunt of the proband (N09 2223), was 36 years old when examined. At 18 months of age, she developed febrile *status epilepticus* without later motor deficit but secondary psychomotor decline. When she was 10, she developed afebrile GTCS and transient losses of consciousness (suggesting either absences or partial seizures). She always had school difficulties and developed psychotic manifestations. At the time of the study, she presented with moderate intellectual deficiency. EEGs during childhood were unavailable but those during adulthood were normal. MRI was normal. During childhood, she was treated with PB which appeared effective. In adulthood, carbamazepine (CBZ) was ineffective but she finally became seizure-free under VPA. She was still treated with VPA at the time of the study.

Family 2 was of polish Ashkenazi origin on the paternal side. There was no family history of seizures or cognitive impairment in the previous generations. The three patients were born after uneventful pregnancies and had normal neonatal periods. None of them had psychiatric features and neurological examination always remained normal. Patient N09 2853 was 24 years old at the time of the study. Since the age of 7 months, seizures occurred in clusters lasting a few minutes, with symptoms consisting of sudden cry and behaviour changes. There were one or two clusters per months, frequently favoured by hyperthermia. Brain MRI, at 5 years of age showed a frontal median dermoid cyst. She underwent resection of the cyst without any modification of seizure frequency. From the age of 9 years, the seizure semiology was modified, starting with a short yell or the repetition of a word related to the events of the previous days and followed by hyperactivity, fear, pallor and tachycardia. Consciousness was not altered during the shorter episodes. Clusters including more than 10 daily seizure episodes occurred over 2 to 3 days with intervals of 6 weeks to 3 months. They occurred mostly during daytime or as she fell asleep and were currently triggered by fever, emotions or sleep deprivation. She frequently experienced right frontal headache during the days preceding the seizures. At the age of 10 years, stereoencephalography showed rapid spikes followed by slow spike-waves in the left frontal cortex with contralateral propagation. The diagnosis of cryptogenic partial frontal epilepsy was considered. She has been treated with CBZ from the age of three years to the age of nineteen years in addition to other successive drugs (VPA, VGB, LTG, TPM and LEV). Seizure offset occurred when she was 19 years old, as she was treated with CBZ and LEV, whose doses were then progressively decreased. Neuropsychological testing, at the age of 16 years showed a VIQ score of 115 and a PIQ score of 87 with slight impairment in working memory and visual treatment. She is now 23 years old and is attending the courses of French engineering school. Patient N09 2265 was one year younger than her sister. She experienced a first cluster of GTCS lasting one hour, when she was five years old, 12 hours after a febrile episode. At 6 years, she had two similar clusters favoured by hyperthermia as well as several afebrile nocturnal episodes. Neuropsychological testing, when she was 7 years old showed a VIQ score of 117. She continued to experience repeated clusters of seizures with a modified semiology including automatic gestures, chewing and elevation of the left arm. Seizures were mainly triggered by fever. Some nocturnal tonic-clonic seizures also occurred. She was first treated with VPA, which was substituted with CBZ at the age of 10 years. Treatment could be stopped 6 years later. Now aged 22 years, she attends the classes of a French engineering school as her sister. The youngest sister (N09 2851) was 15 at the time of the study. Since 9 months of age, she experienced clusters of GTCS during febrile episodes as well as nocturnal seizures with a semiology consisting of straightening up of the body, incoherent language, hypertonia with hand contractions, deviation of the head (mainly on the right), chewing and bruxism. Seizure semiology was progressively modified with staring, back dropping and yelling, clonic movements followed by hypertonia with ocular revulsion and chewing. EEG was in favour of a possible fronto-temporal origin. She has first been treated with CBZ. LTG was added when she was 7 years old and CBZ was switched to LEV two years later. Seizure offset occurred at 13 years of age. When seen for the last time, she was attending normal school although she displays slight reduced processing speed. She has won several championships of bridge.

Family 4, of French origin, included 3 female patients who all had GTCS but detailed clinical data were available for the proband only (N09 0418). She was born after uneventful pregnancy and had a normal development before the onset of seizures. She experienced a first brief generalized febrile seizure at 13 months. Two weeks later, she had a cluster of brief GTCS without fever and a treatment by VPA and Clonazepam (CLN) was started. At the age of 3 years, she had several generalized seizures in clusters. Seizures were brief (< 2 minutes) but repeated over several days (3-4 days). Afterwards, she had occasional episodes of GTCS occurring in cluster, which were commonly triggered by fever and lasted one or two minutes or even only a few seconds. VPA was replaced by TPM for a better control of the seizures. She never had myoclonia or partial seizures. At the age of 6 year, her language was delayed but she was able to make simple sentences. She was described as an introverted child, who had difficulties to establish social interactions. She was capable of communicating with words but did not use language spontaneously. For instance, she preferred pointing at objects or images rather than naming them. When examined for the last time, at 7 years old, she had a mild cognitive and language delay. Her mother was reported to have generalized seizures between age 2 and 6 years, that were first treated by PB and later on by VPA. At that time, poly spike-wave discharges were observed on EEGs. At the age of 18 years, she became free of seizures without treatment. However, when examined at age 44, she presented with mild intellectual disability. The half-sister of the proband had 3 “Grand Mal” seizures at the age of 5 years old without fever. She became free of seizures when treated with VPA. However, during the adolescence, she presented with important scholar difficulties and psychiatric instability.

Family 12 includes two affected sisters born from non consanguineous parents of French origin. Both sisters have had intra-uterine growth retardation related to a homozygous variant of the methylenetetrahydrofolate reductase gene in the mother. However, they were considered healthy full-term newborns. The proband (N08 0696), who was 4 years old at the time of the study, experienced her first seizures at 11 months during a febrile otitis episode. She had about 10 febrile GTCS lasting a few minutes the same day with normal examination in-between, normal interictal EEG and normal brain MRI. Another series of afebrile paroxysmal events, characterized by numerous absences without abnormal movements lasting less than a minute, occurred one week later despite the initiation of VPA therapy. CLB was therefore added to VPA. Three more clusters of 5-10 seizures occurred at the ages of 2, 3 and 4 years, requiring an add-on treatment with LTG. The clinical manifestations were heterogeneous, including absences, clonic and tonic seizures, sometimes associated with eyes turning. Most seizures lasted 1 to 5 minutes, but clusters of 10 to 15 minute-long seizures were also observed. Interictal EEGs remained normal except for occasional posterior slow waves. At the age of 3.5 years, psychomotor retardation and language delay became evident. She also had a mild microcephaly (-2 SD). The youngest sister (N 09 1103) was 2.5 years old at the time of the study. She had a first cluster of ∼10 afebrile GTCS at 8.5 months, leading to a treatment by VPA. Brain MRI was normal and interictal EEG revealed bitemporal slow waves but no paroxysmal activity. Another seizure cluster occurred a few days later and VGB was added to VPA. Two more episodes of 8-10 afebrile seizures occurred at the age of 21 months and were characterized by generalized tonic manifestations, sometimes asymmetrical with head turning, lasting a few minutes. EEG recording of a seizure showed generalized discharges from the beginning of the seizure. After replacement of VGB by TPM, no other seizures occurred. Her psychomotor development was slightly retarded, as she sat alone at 13 months, walked independently at 18, but her speech ability was relatively preserved at the age of 2. Her head growth followed the -2 SD curve until 6 months then slowed down to -2.5 /-3 SD.

Family 17 was of French origin and included a single affected female patient (N07 0897) who was 7 years old at the time of the study. Her parents were reported to be healthful and there was no further history of febrile seizures or epilepsy in the family. The proband was born after an uneventful pregnancy and had a normal development before the onset of seizures. At 15 months, while she was febrile, she started having clusters of brief generalized tonic seizures that required hospitalization. A treatment by VPA was set up but tonic seizures lasting 2-3 minutes and repeating during several days recurred at 19 months in a febrile context. Despite the successive introduction of multiple AEDs (CLB, TPM, PB and PHT), brief hypertonic seizures occurring in clusters persisted over time. An EEG performed at 19 months was normal. At 5 years old, she had a normal neurologic examination but showed a global developmental delay predominating in language and learning (estimated to a one-year delay).

The proband of family 18 was born at 36 weeks from a bichorionic gemellar pregnancy. Her parents, her twin sister and a youngest sister were all healthy. Her psychomotor development was normal until the onset of seizures. She experienced a first afebrile generalized convulsive *status epilepticus* at the age of 14 months. Investigations including brain imaging, lumbar puncture, toxic and metabolic screenings were normal. EEG was normal. She was treated with CLN and VPA. She started walking at 16 months, almost two months after her twin sister. She had new episodes of febrile GTCS at 1.5, 2.5 and 3.5 years. Behavioural disturbances and language delay became obvious around 3 years old. A decrease in CLN dosage at the age of 4 years led to a new *status epilepticus* and LTG was added to CLN and VPA. Likewise, a decrease in VPA dosage at the age of 5 years was unsuccessful. From the age of 5 years, she had several clusters of GTC seizures, most of them triggered by mild fever. At the age of 7 years, she had a moderate cognitive impairment including a global executive dysfunction. Karyotyping was normal and search for X-Fragile expansion was negative. Diurnal and nocturnal EEGs and Brain MRI remained normal. A novel attempt to decrease CLN dosage at 7 years led to the recurrence of afebrile seizures in clusters. CLN was replaced by CLB. At the time of the study (11 years), she was free of seizures since age 9 on tritherapy (VPA, CLB, TPM). She was able to read and her language appeared normal. However, she had major behavioural and personality disturbances. She also had difficulties with logical reasoning and graphics. She never had ataxia or myoclonic jerks and her seizures were photoinsensitive.

## DISCUSSION

In this study, we report 15 novel point mutations and three deletions in the *PCDH19* gene in 18 unrelated female patients, for an overall mutation frequency reaching 16% (18/113). We also tested 37 male patients in this study but no mutation was found. However, we cannot exclude that mosaicism has been missed by screening only DNA extracted from blood cells in these patients.

Although all the previously reported mutations were clustered in exon 1 of *PCDH19* (corresponding to the extra-cellular cadherin domain of protocadherin 19) (Depienne, et al., 2009; Dibbens, et al., 2008; Hynes, et al., 2009), we now report a nonsense mutation in exon 4 (p.Arg886X) that segregated in several affected members of family 1. This shows that the *PCDH19* mutational spectrum is not limited to mutations in exon 1. Furthermore, two whole gene deletions and one partial deletion were identified using quantitative real-time PCR. These deletions constitute the first micro-rearrangements involving *PCDH19* identified in female patients with febrile seizures and epilepsy. Altogether, these results confirm that mutations in *PCDH19* are associated with a loss-of-function of protocadherin 19 in cells that have inactivated the normal X chromosome, and that screening of *PCDH19* should include a specific method to search for rearrangements in addition to direct sequencing of the whole coding sequence.

As previously reported, the phenotype of the patients with *PCDH19* mutations was highly variable between families but also within affected members of a same family (Table 2). This variability includes the age at onset (mean age at onset: 16 months, ranging from 7 months to 5 years), the occurrence of *status epilepticus* episodes (42%, 10/24), the response to AEDs (pharmacoresistance in 76% of the cases, 19/25) and the temporal evolution of the patients. However, seizures occurring in cluster (92%, 23/25 patients) and a high sensitivity to fever (90%, 20/22 patients) were almost invariably present in PCDH19-positive patients. All patients have had generalized seizures (although in some cases, the generalized seizures were only in febrile contexts) and 68% exhibited multiple seizure types (17/25) including partial seizures (of frontal or temporal origin) and atypical absences. Although seizures appeared highly resistant to AEDs during the first years of life, the frequency and pharmacoresistance of seizures tended to decrease over time, and some patients were even free of seizures during adolescence or adulthood on monotherapy. Myoclonic jerks were infrequent in patients with *PCDH19* mutations (9%, 2/23) and photo sensitivity was reported in only one case out of 25 (<5%). In contrast, a high frequency of behavioral disturbances, including autistic features, was noticeable (28%, 7/25 patients). In some patients, social withdrawal or personality troubles were even the most prominent and disabling feature when the children became older. Interestingly, the phenotypes of the patients with *PCDH19* deletions seemed indistinguishable from those with point mutations although this finding needs to be confirmed with a larger number of patients carrying deletions.

Intellectual deficiency was also very variable from one patient to another: cognitive impairment was reported to be mild in 6 patients, moderate in 10, and moderate/severe in 2 patients. One patient was too young to evaluate her cognitive status (family 7). Interestingly, in families 1 and 2, most or all patients presented without cognitive impairment. In family 2 for instance, all the 3 sisters with the p.Thr146Arg mutation had normal cognitive abilities. The history of epilepsy in the 3 sisters was nevertheless similar to other patients with moderate mental retardation and included in particular many seizures in cluster during childhood, persistence of the seizures despite polytherapy and later development of partial seizures of frontal or temporal origin. This highly suggests that the cognitive prognosis is not related to the severity of epilepsy. In family 1, the history of febrile and afebrile seizures in the proband, her mother and her grand-mother was highly suggestive of Generalized Epilepsy with Febrile Seizures plus (or Genetic Epilepsy with Febrile Seizures plus, GEFS+; MIM# 604233) (Scheffer and Berkovic, 1997). It is unclear so far whether the favorable cognitive outcome in these patients is due to the mutation itself, which could have milder functional consequences on the protocadherin 19. The large intrafamilial variability of the phenotype (in particular in family 1) however suggests that other genetic or non genetic modifier factors could be involved as well. One attractive hypothesis is that this phenotypic variability could be due to random or skewed X inactivation. Cellular interference is based on the random X inactivation resulting in a functional mosaicism in females (Compagni, et al., 2003; Depienne, et al., 2009; Johnson, 1980; Wieland, et al., 2004). A totally skewed X inactivation in neurons would theoretically leads to a homogenous cell population (expressing or not the normal protocadherin 19), corresponding to a non-pathogenic situation. It is possible that a partially skewed X inactivation (such as 60:40, 70:30 or 80:20) could represent situations less severe than a random X inactivation (50:50) where the cellular interference is expected to be the highest. The severity of the epilepsy could therefore be correlated with the relative amount of inactivated neurons for each chromosome in the brain and some mutation carriers, like mutated mothers in family 13 and 14 for example, could even be asymptomatic due to totally skewed X inactivation. However, we cannot exclude that the missense mutations in these families correspond to rare benign variants.

The wide phenotypic expression associated with *PCDH19* mutations is very reminiscent of what is observed for patients with *SCN1A* mutations. It is now well-established that *SCN1A* mutations are associated with a large clinical spectrum ranging from febrile seizures only to typical or atypical Dravet syndrome (Depienne, et al., 2010; Harkin, et al., 2007). We have previously reported that the similarity between *PCDH19* and *SCN1A* clinical spectra comprises Dravet syndrome; now, we show that it also extends to that of GEFS+. Indeed, intellectual disability as well as partial epilepsy can also be part of *SCN1A*-positive GEFS+ families (Depienne, et al., 2010; Escayg, et al., 2000). Differentiating one condition to the other would therefore be difficult in some cases. However, some clinical features could help prioritizing the analysis: for instance female patients with an age at onset after 12 months and presenting with seizure clusters should be first screened for *PCDH19* mutations, especially when the families include several females but no male patients. Regarding Dravet Syndrome, screening for *PCDH19* mutations should be performed for female patients when analysis of *SCN1A* (including a method searching for *SCN1A* micro-rearrangements) is negative.

Given the unusual mode of inheritance and the wide phenotypic variability associated with *PCDH19,* genetic counseling appears delicate. In the case of mutations inherited from an asymptomatic father, all the daughters are expected to be affected. Therefore, taking into account the frequent bad cognitive prognosis, it could be possible to offer a prenatal diagnosis using only a fetal sex determination from maternal blood (Wright and Burton, 2009). Female patients with *PCDH19* mutations have a 50% risk to transmit the mutation but only the females would be affected, for a global risk of 25%. However, in some situations, genetic counseling could be even more difficult, such as in family 17 in which the proband had a *PCDH19* deletion encompassing other nearby genes with a recessive X-linked inheritance such as SRPX2. In that case, the patient will have a 25% risk to have an affected daughter and of a 25% risk to have a son affected with a phenotype of variable severity including rolandic epilepsy, oral and speech dyspraxia, mental retardation and perisylvian polymicrogyria.

In conclusion, these results extend the mutational and clinical spectra associated with *PCDH1* 9-related epilepsies and show that mutations in *PCDH19* are a frequent cause of epilepsy in females and should be considered even in absence of family history and/or mental retardation.

## Supplemental material

**Supp. Figure S1.** Pedigrees of the families and segregation analysis of the *PCDH19* mutations in families 3 to 15. Squares represent males, circles females; Upper right black corner: Generalized seizures; Upper left black corner: Partial seizures; Lower right black corner: FS; Lower left black corner: intellectual disability. Right black half (mother of the proband, family 10): Febrile seizures (FS) and tonic clonic seizures in childhood, normal cognitive abilitie; Grey symbols: Epilepsy of unknown type. Dots in the middle of the squares indicate unaffected mutation carriers. The arrows indicate the index cases. B) Sequence electrophoregrams of the mutations. The mutation nomenclature is based on the *PCDH19* transcript reference EF676096.

**Supp. Figure S2.** Orthologous and paralogous protein alignments, showing the high conservation of each amino-acid altered by missense mutations in vertebrates and in the delta protocadherin paralogous genes.
