# Supplementary figures and images for: Mutations and Deletions in PCDH19 Account for Various Familial or Isolated Epilepsies in Females
Source: Hum Mutat. 2011 Jan;32(1):E1959–75. doi: 10.1002/humu.21373 (PMC3033517; doi:10.1002/humu.21373)

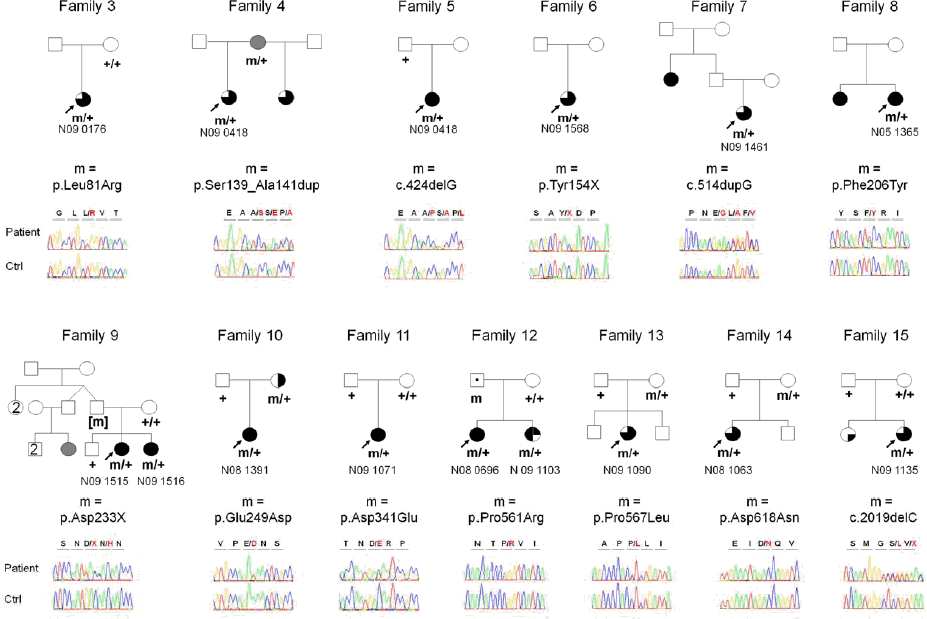

Supplement: Supplementary file 1 [file humu0032-E1959-SD1.gif]

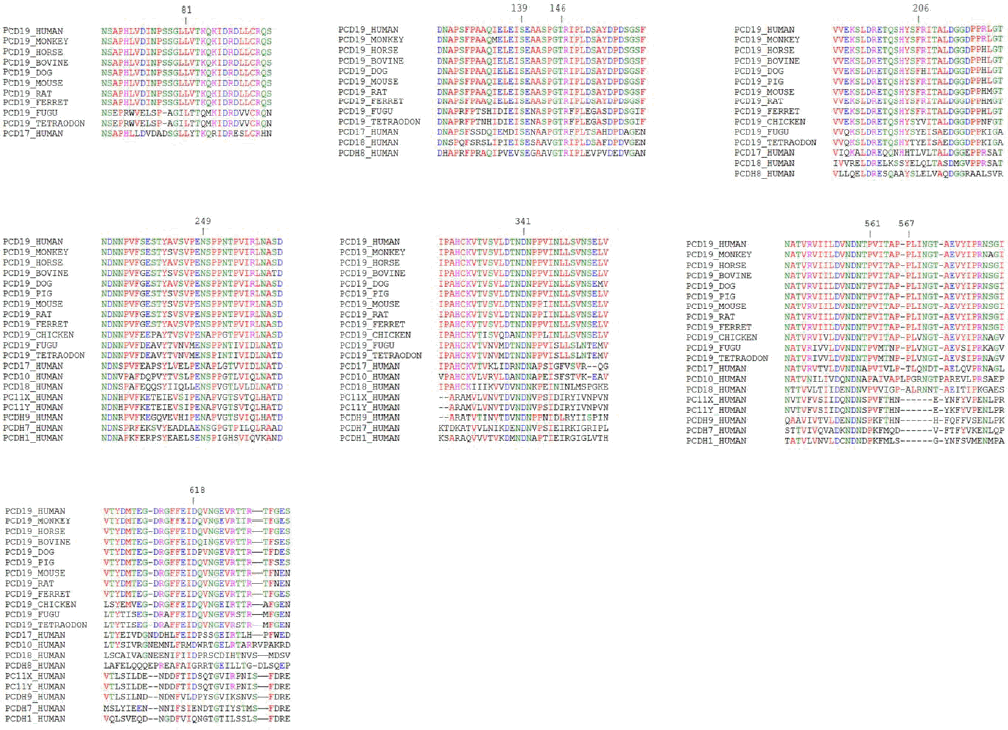

Supplement: Supplementary file 2 [file humu0032-E1959-SD2.gif]
